# Precocious puberty under stressful conditions: new understanding and insights from the lessons learnt from international adoptions and the COVID-19 pandemic

**DOI:** 10.3389/fendo.2023.1149417

**Published:** 2023-05-02

**Authors:** Maria Elisabeth Street, Davide Ponzi, Roberta Renati, Maddalena Petraroli, Tiziana D’Alvano, Claudia Lattanzi, Vittorio Ferrari, Dolores Rollo, Stefano Stagi

**Affiliations:** ^1^ Department of Medicine and Surgery, University of Parma, Parma, Italy; ^2^ Unit of Paediatrics, P. Barilla Children’s Hospital, Azienda Ospedaliero-Universitaria of Parma, Parma, Italy; ^3^ Department of Pedagogy, Psychology and Philosophy, University of Cagliari, Cagliari, Italy; ^4^ Health Sciences Department, University of Florence, Florence, Italy; ^5^ Azienda Ospedaliero Univesitaria Meyer IRCCS, Florence, Italy

**Keywords:** central precocious puberty, adoption, COVID-19, rapidly progressive precocious puberty, neurobiology of puberty, psychology

## Abstract

Neuro-biological variations in the timing of sexual maturation within a species are part of an evolved strategy that depend on internal and external environmental conditions. An increased incidence of central precocious puberty (CPP) has been described in both adopted and “covid-19 pandemic” children. Until recently, it was hypothesised that the triggers for CPP in internationally adopted children were likely to be better nutrition, greater environmental stability, and improved psychological wellbeing. However, following data collected during and after the coronavirus (COVID-19) global pandemic, other possibilities must be considered. In a society with high levels of child wellbeing, the threat to life presented by an unknown and potentially serious disease and the stressful environment created by lockdowns and other public health measures could trigger earlier pubertal maturation as an evolutionary response to favour early reproduction. The main driver for increased rates of precocious and rapidly progressive puberty during the pandemic could have been the environment of “fear and stress” in schools and households. In many children, CPP may have been triggered by the psychological effects of living without normal social contact, using PPE, being near adults concerned about financial and other issues and the fear of getting ill. The features and time of progression of CPP in children during the pandemic are similar to those observed in adopted children. This review considers the mechanisms regulating puberty with a focus on neurobiological and evolutionary mechanisms, and analyses precocious puberty both during the pandemic and in internationally adopted children searching for common yet unconsidered factors in an attempt to identify the factors which may have acted as triggers. In particular, we focus on stress as a potential factor in the early activation of the hypothalamic-pituitary-gonadal axis and its correlation with rapid sexual maturation.

## Introduction

Central precocious puberty (CPP) is defined as the appearance of physical and hormonal signs of pubertal development at an earlier age than considered normal. In girls it is defined by the appearance of secondary sexual characteristics before the age of 8 years and is caused by a premature activation of the hypothalamic-pituitary-gonadal axis (HPG) (1). The average age at onset of puberty may vary in some subpopulations. For instance, in African American and Hispanic American girls, thelarche may normally occur at 7 years of age, and menarche occurs approximately 0.5 years earlier than in Caucasians. The estimated prevalence of CPP is approximately 1 in 5,000–10,000 among Caucasians, with a higher incidence in girls than in boys (approximately 10:1), on a worldwide basis (2). The incidence of central precocious puberty has been steadily increasing over the last century, with the average age at menarche dropping from 17 years in the early-1800s to 13 years by the mid-1900s, with a further minor decline over the last three decades (3). Danish data for the 20-year period from 1998 to 2017 showed a 3-fold and 2-fold increased incidence of CPP in girls and boys, respectively (4).

This trend towards earlier pubertal onset – the “secular trend of puberty” – is related with both genetic and environmental causes (3, 4).

Decreased infant mortality due to increased caloric resources, antibiotics and improved medical care is often reported as one of the main factors driving the earlier onset of puberty observed across industrialized countries. Yet, increasing amounts of data also support a “psychosocial acceleration hypothesis” (5, 6) that maintains that early life adversities speed up sexual maturation, especially in populations where nutritional stress and mortality rates are low (7). We suggest that the hypothesis that early life adversities affect pubertal timing is supported by studies on international adoption and, recently, by the surge in reported CPP during the COVID19 pandemic.

International adoption from developing countries is a widespread phenomenon both in Europe and North America. Although a decrease has been observed in the last twenty years (-81.7% from 2004 to 2018), numbers remain high (8). In 2021, Italy registered a national average of 7.3 adoptees for every 100,000 residents in the paediatric population. The country is second only to the United States for international adoptions and the average age that adopted children have at arrival in Italy is approximately 7 years (9). Adoption is one of the most widely recognized risk factors for CPP (2, 10, 11) although the causal relationship between adoption and CPP is still a matter of debate, and the mechanisms remain elusive. Current speculation focuses on the influence of emotional and environmental factors. Among the latter, early-life nutritional deficiency followed by increased adiposity after adoption has been suggested as a potential trigger (12). However, most of these hypotheses do not apply to girls presenting with CPP during the covid-19 pandemic (13). Different hypotheses have been put forward, suggesting both direct and indirect stimulating factors (13, 14). However, the underlying mechanisms are uncertain and no one factor can explain the dramatic increase in CPP observed during the covid-19 pandemic in girls.

This review presents an overview of the possible triggers for early pubertal onset both in internationally adopted children, and in children during the pandemic period, calling for an urgent evaluation of the psychosocial acceleration hypothesis.

## The evolutionary life history of puberty

Reproductive development is regulated by the hypothalamus-pituitary-gonadal (HPG) axis. This consists of three main anatomical areas that are sequentially and bi-directionally linked to each other: the arcuate nucleus (infundibular nucleus in primates) of the hypothalamus, that contains gonadotropin releasing hormone (GnRH) neurons; the adenohypophysis, that contains the gonadotrophs; and the gonads. Across mammalian species the physiological steps necessary for successful reproduction, i.e. sexual maturation and fertility, require an increase in the pulsatile activity of GnRH neurons (15–17). When GnRH is released intermittently and within a specific amplitude range it stimulates the secretion of LH and FSH in the portal system (18). In humans and non-human primates GnRH neurons stimulate LH secretion during infancy and at the beginning of puberty. Between these two developmental periods GnRH neurons show a very low intermittent activity, secrete very small amounts of hormones, and LH, FSH and sex steroids in the blood are found at their lowest concentrations (19, 20).

This on-off-on neurosecretion of GnRH is not present in rodents and is associated with the juvenile developmental stage typical of primates (20). The juvenile life stage, which further delays pubertal onset, is characterized by a slow growth that results from a trade-off between the metabolic requirements for developing a large, costly brain and those for body growth. Peak brain metabolic consumption occurs at the age of 4-5 years in humans, when the brain’s metabolic requirements equal 66% of resting energy expenditure (21, 22) and then fades as brain development moves from growth toward synaptic pruning. This is also the slowest period of annual growth rate in terms of fat deposition and body length in humans (23). Once the brain metabolic consumption tapers off, energy supply is redirected toward somatic, maintenance and reproductive development. However, in our species a further lengthening of the juvenile stage is observed, characterized by a mid-spurt growth, an adiposity rebound and by the first sign of pubertal development expressed as axillary and/or pubic hair growth (24).

Despite the evident advantages of growing a larger body or a bigger and complex brain, there are also obvious risks in delaying the onset of reproduction, the most serious of which would be dying before reproducing. Accordingly, the timing of reproductive readiness is crucial for any organism. Each species has evolved a reproductive clock which responds to environmental cues that throughout the species’ evolutionary history have been relatively predictable. Yet variations in the onset of sexual maturation are observed within species, between sexes and across generations and individuals. For example, in humans the age of pubertal onset has a range of variation of approximately 4 years, with girls entering puberty earlier than boys and children from non-industrial populations showing the most delayed onset (25). It is possible that variations in the timing of sexual maturation within a species are, at least in part, an evolved conditional strategy that depends on internal and external environmental conditions (5, 26, 27).

**Figure 1:**
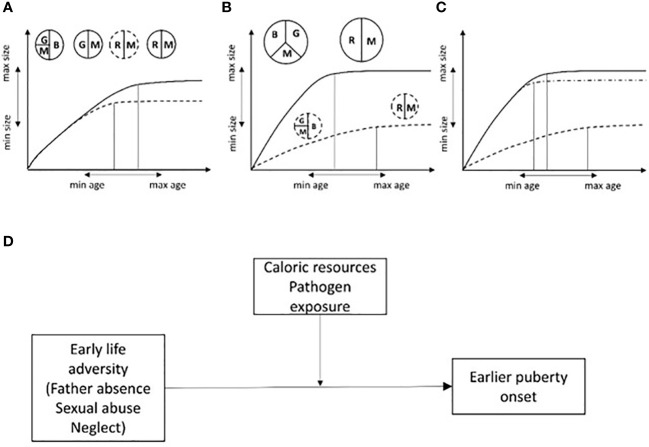
Somatic growth vs reproduction trade-offs in energy allocation are expected to influence the relationship between body size and age at sexual maturity. **(A)** Two individuals with same genotype are exposed to the same caloric resource regimen and pathogen load. Resources will be allocated toward brain and somatic growth and maintenance (immune system). If for some reason (i.e. a new mutation) one of them matures earlier (dashed line), resources will be allocated to reproduction at the expense of somatic growth. **(B)** Two individuals with the same genotype experiencing different environmental risks (different levels of caloric intake, pathogen load and thus high vs low mortality/morbidity risk). Individuals with less resources will take longer to develop, coming near the latest species-specific limit for sexual maturation but reaching the lower limit for species-specific adult body size (dashed line). On the contrary, the individual with better resources can afford to invest both in brain development, body growth and maintenance, therefore, maturing earlier and becoming bigger (secular trend). **(C, D)** If conditions threatening child survival/well-being are present, the extent to which these conditions will influence sexual maturation will depend on caloric resources and pathogen load (i.e. the amount of resources available for somatic growth, maintenance and reproduction). In poor environments, psychological and social threats are expected to have a small and negligible effect on sexual maturation while in resource-richer conditions higher adversity is predicted to speed up sexual maturation relative to conditions of low psychosocial threat. This is represented by the dashed-dotted line in **(C)** and by a moderation model in **(D)**. G, somatic growth; B, brain growth; M, maintenance; R, reproduction. Size of circles and slices are proportional to the amount of resources available for the development of different organismal systems. Double arrows indicate the potential span for species-specific age and size at sexual maturity (hypothetical scale). Size of circles, slices and or double arrows are arbitrary.

Models of life history theory provide evidence for two important selective pressures that govern the timing of sexual maturation in a species. These are: a) “extrinsic mortality” which represents any environmental condition that causes disease and increases the risk of mortality; a higher risk of adult extrinsic mortality should result in an anticipation of reproductive age, and b) juvenile mortality which when high should result in a postponement of reproductive onset (28). SSafer and nutritionally richer environmental conditions during early development are expected to speed-up growth and sexual maturation, resulting in organisms that are larger (and thus more competitive and, possibly, with higher fertility) at sexual maturity. In calorie deprived and harsh conditions, growing more slowly, in order to wait for better times to come, could be the best strategy but it would result in an individual of smaller size at sexual maturity. These models fit well with secular trends in human pubertal development (28, 29) (**Figure 1**).

### Early life adversity and puberty

While there is consensus on the role played by energetic stress in postponing reproductive development (30), the effects of psychosocial stress on the onset and tempo of puberty are less clear. Lower socio-economic status has been found to predict earlier sexual maturation through mechanisms that do not depend on body mass index (BMI) (31, 32). One frequently reported finding is that psychological stress, experienced during early childhood, accelerates pubertal development, a phenomenon termed “psychosocial acceleration” (33). Stressors that represent an explicit threat for child survival (domestic and urban violence; environmental catastrophes) or that result in child trauma (sexual abuse) are associated with early pubertal development (34). However, there is not yet a consensus on the causative link. While accepting a hierarchical order of importance between nutritional and psychological stress on pubertal development (7, 35), some authors assume that environmental conditions linked to extrinsic mortality, experienced during early childhood, if not successfully buffered, for example by parental protection or other means to reduce the (perceived) mortality risk, will translate into faster reproductive development (36). Importantly, for protection strategies to work the child must not be explicitly aware of risks to life expectancy (37, 38).

Many evolutionary psychologists consider environmental harshness, for example social threats or deprivation as an important factor for speeding up sexual maturation (36). Most of the evidence indicates that stressful and unsupportive family environments are associated with earlier maturation in girls with an anticipation in particular of age at menarche (39). In general, family breakdown, especially when linked to socially deviant behaviour in fathers, seems to be a strong predictor of early menarche (40). Some studies have shown that maltreatment and poor maternal care are also variables implicated in early menarche (41). A recent review by Pham et al. (42) revealed that family structure and functioning, in particular the absence of the father, predicts early puberty (EP) in females. Belsky et al. confirmed this but in high income families only (43). The environmental variables identified as affecting the timing of puberty in girls and boys vary across studies. In general, the literature highlights that early puberty in girls correlates to adverse family circumstances related to poor parenting behaviour such as a lack of emotional warmth, overly strict maternal attitudes and conflict between parents. Clearly, the effects of family environment in early in life on pubertal timing are multifaced and complex to investigate. Overall, the most significant predictors of early menarche reported in the literature are harshness during the first 5 years of life, with an absent father and sexual abuse (6, 44) playing a major role whereas secure attachment to the maternal figure is recognized as a protective factor capable of supporting girls in stressful adverse contexts (45, 46).

Clearly, nutritional stress has different developmental outcomes compared to psychosocial stress. To some extent, this is not surprising because i) different types of stressors act through different neurobiological mechanisms; ii) if the organism is energetically ready to invest in reproduction, then under a higher risk of extrinsic mortality (indirectly experienced during development through harsh environments) it may pay to accelerate pubertal development (35, 36: **Figure 1**).

In the next paragraph, the components of the neuroendocrine system known to integrate environmental cues related to resource availability and social conflict (physical and psychosocial stress) into the HPG axis to modulate pubertal onset will be described.

## Neurobiological mechanisms linked to pubertal development

The ability to integrate environmental cues in the HPG axis depends on central and peripheral neuroendocrine factors that, by conveying environmental, metabolic and homeostatic information modulate GnRH neurosecretion. Some of these factors act directly on the GnRH neurons, while others mediate their effects through kisspeptin neurons of the arcuate nucleus, the actual GnRH pulse generators. Loss of function mutations in the kiss1 gene or its receptor GPR54 have been consistently found to result in the absence of puberty in humans and animals (47, 48).

The release of kisspeptin is intermittent and highly correlated with the intermittent neurosecretion of GnRH (49) and the infusion of kisspeptin in prepubertal male monkeys induces GnRH intermittent secretion (50, 51). Moreover, kisspeptin levels in the hypothalamus of monkeys increases as animals move from the pre-pubertal to pubertal stage and the expression of kisspeptin and its receptor GPR54 are higher at the onset of puberty compared to prepuberty in both rats and monkeys (52). These neurons co-express two peptides along with kisspeptin: neurokinin B (NKB) and opioid dynoprhin (DYN) and the coordinated activity of these neuromodulators determines kisspeptin release.

Stimulatory regulation of GnRH neurons derives also from glutamatergic neurons scattered throughout the hypothalamus, norepinephrine and from another population of kisspeptin neurons found in the anteroventral periventricular nucleus (AVPV) of the hypothalamus (the preoptic area of primates) that are important for the LH surge that precedes ovulation (47, 52). Glutamate is the main excitatory neurotransmitter in the brain and glutamate receptors are found on GnRH and kisspeptin neurons. Blocking NMDA receptors suppresses GnRH pulses and the preovulatory surge of LH (47). The excitatory effects of glutamate on GnRH neurosecretion have been reported in all three major models of puberty where antagonists of glutamate delay puberty while agonists anticipate it. The concentration of glutamate in the hypothalamus increases during the juvenile period and reaches a maximum after puberty onset (53) and peaks can be observed during the preovulatory LH surge.

Catecholamine noradrenaline has been extensively studied. Norepinephrine is released centrally from three areas of the brainstem and when it binds to alpha2- and beta-receptors it has stimulatory effects on LH secretion (52). The pharmacological depletion of central catecholamines in prepubertal rats resulted in delayed vaginal opening (54, 55) and the oestrogen dependent role of NE in the preovulatory surge of LH is well known in rats and primates (56). Moreover, there seems to be a close positive correlation between noradrenergic content in the hypothalamus and pubertal development: noradrenaline levels are higher in the hypothalamus of rats with precocious puberty and on the day of vaginal opening compared to prepubertal animals (57). Similar results have been reported for rhesus macaques (58). A metabolomic study reported a higher level of NE metabolites in urine samples of girls with central precocious puberty compared to controls (59). These data support the hypothesis that catecholamines play a permissive role in the regulation of pubertal development.

Inhibitory regulation of GnRH occurs through direct or indirect (kisspeptin suppression) innervation of GnRH neurons (52, 60). GABA is the main inhibitory amino acid neurotransmitter that derives from the conversion of glutamate through the activity of glutamic acid decarboxylase (GAD). Experiments on monkeys by the Terasawa’s group have clearly showed that GABA has inhibitory effects on the GnRH pulse generator (48).

Treatment of monkeys with GABA or with the GABA antagonist bicuculline resulted in precocious puberty and although mutations within the GABA system do not affect puberty, the GABAergic pathway has also been implicated in a recent GWAS study on early menarche (61) (52). Terasawa’s *in vivo* studies on non-human primates provided support for a model in which during the prepubertal life stage the GnRH pulse generator is under tonic GABA inhibition, possibly through the action of GABAergic activity on kisspeptin neurons (62). This tonic inhibition decreases throughout pubertal development leading to a higher glutamate/GABA activity ratio and kisspeptin release onto GnRH neurons.

### Nutrition dependent regulation of pubertal onset

Environmental cues that convey information on energetic resources and environmental risks must be processed and integrated within a network of hypothalamic nuclei that modulate reproductive development (27). Kisspeptin neurons of the arcuate nucleus are targets of hypothalamic nuclei that convey information about metabolic and psychosocial stressors. These neurons play a key role in the integration of metabolism and reproduction as they directly or indirectly receive inputs from hormone and peptides linked to energetic homeostasis. Among these, the adipokine leptin has been considered a key modulator of pubertal growth. Leptin is released from adipocytes in the circulatory system to relay information of fat mass and energy status to the hypothalamus and, since its discovery, has been under scrutiny as a trigger for puberty. By acting through GABA-ergic, POMC and AgRP/NPY neurons that target kisspeptin neurons in the arcuate nucleus (63) leptin may have an important role in pubertal development. A role of leptin in puberty onset has been reported in mice and rats (52). Leptin injected in prepubertal monkeys increases LH and oestradiol and causes premature menarche (64) but data are controversial in humans (for reviews see 52, 65). Leptin administration to human subjects with leptin deficiency influenced the age of pubertal onset (66). Indeed, leptin levels rise during the prepubertal period (67). It can be hypothesised that by keeping track of overall energetic status, leptin may convey information on the anabolic status of an individual during mid childhood. This is supported by the observation that in prepubertal children leptin correlates with DHEA levels and that DHEA levels correlate with protein consumption and larger increases in body mass (68). Moreover, the adiposity rebound observed during adrenarche correlates with levels of leptin and insulin-like growth factor 1 (24). Since higher BMI during childhood predicts early menarche, the current consensus is that leptin has a permissive role in the activation of the GnRH pulse generator.

Conditions leading to a negative energy balance suppress GnRH outflow and produce lower expression of kisspeptin (69). Kisspeptin neurons receive inputs from POMC and AgRP/NPY neurons. POMC neurons of the arcuate nucleus express receptors for leptin, NPY and insulin (70). POMC is a precursor for α-melanocyte stimulating hormone (α -MSH) and β-endorphin. α -MSH stimulates LH secretion in humans (71) and blocks the two α -MSH receptors (MC3R and MC4R) by AgRP causes infertility in mice (72). Central melanocortin appears to be an important mediating link between leptin and GnRH and kisspeptin neurons (73, 74). In monkeys, hypothalamic NPY and its receptor NPY1 are more expressed while kisspeptin and its receptor are less expressed in prepubertal than in pubertal and adult males (75), providing some support to the hypothesis that NPY may act as the break for the GnRH pulse generator (20). AgRP/NPY neurons also coexpress GABA and the action of these three peptides stimulate feeding behavior by, in part, inhibiting the POMC pathway.

### Stress dependent regulation of pubertal onset

One of the strongest modulators of the HPG axis is the hypothalamus-pituitary-adrenal (HPA) axis, that modulates the stress response. The stress response begins within the central nervous system with the synthesis and secretion of the corticotropin releasing hormone (CRH), perhaps the strongest inhibitor of the HPG axis (76, 77). Stressors convey different information and require different responses. This is especially evident in the way metabolic and psychological cues of stress activate different central peptidergic pathways: immunological/metabolic stressors inhibit the GnRH pulse through the binding of urocortins to the CRHR2 receptors while psychological stressors are mediated directly by CRH (77). The peripheral products of the HPA axis, glucocorticoids (GC), administered in concentrations that reflect those measured during stress suppress the HPG axis (76). The effects of GC on GnRH appear to be indirect, through kisspeptin neurons, as GnRH neurons do not express GC receptors (60). CRH also innervates the locus coeruleus where it stimulates the release of norepinephrine. Acute stressors also stimulate the secretion of epinephrine from the adrenal medulla. In agreement with an overall inhibitory effect of stress on reproduction, sympathetic activation under stressful condition is inhibitory (76, 77). In short, stress has been shown to suppress reproduction in a myriad of studies and species. However, these data are representative of what happens in adults facing different kinds of acute or chronic stressors. The effects of the activation of the stress system during development may not be the same as those observed in adults. Furthermore, stressful experiences have programming effects on the HPG system during development. In rodents, the effects of an early life stress experience shapes reproductive strategies: for example, female offspring of low caring mothers show higher HPA activation to stressors and an earlier onset of puberty (78). It remains to be understood if the opposite effects of psychosocial stress on sexual maturation depend at least in part on nutritional/health status and the extent to which the HPA axis and the sympathetic adrenal medullary system exert different stress mediating effects on the developing HPG axis.

## Precocious puberty in adopted children: current data and hypotheses

An increased occurrence of precocious puberty in international adoptees was first described in 1981 in Sweden, in a case series of 7 girls adopted from India and Bangladesh. All these girls had Tanner breast stage compatible with initial puberty by the age of 7 years and advanced bone age, and they all progressed very fast into menarche by the age of 7.6 years (rapidly progressing precocious puberty).

In 1991, Proos et al. analysed a Swedish cohort of 107 adopted Indian girls who showed a median menarcheal age significantly lower than both the Swedish and the Indian population (79). Following these first two reports, other case reports and case-control studies confirmed this phenomenon in other Western European countries (2, 10, 80, 81).

Virdis et al. and Baron et al. described the onset of early pubertal development respectively in 19 girls and 13 children (10 girls and 3 boys), presenting with a very rapid weight and height catch-up growth after adoption (80). In addition, a French study performed a survey on adoptive families, confirming that the prevalence of CPP in adopted girls was much greater than in adopted boys (44.9% vs 8.6%) (81). Teilmann et al. were the first to calculate that the risk of CPP in internationally adopted children increased 15- to 20-fold compared with Danish-born children (10). This is consistent with a relative risk of CPP of 27.82 subsequently reported in a Spanish cohort by Soriano-Guillén et al. (2). Both studies confirmed that the increased relative risk was higher in girls than in boys. Moreover, the Italian and the Danish authors described that an older age at adoption was associated with an earlier onset of pubertal development in girls (10, 80).

It has also been shown that, prior to clinical signs of puberty, adopted girls showed pituitary-gonadal activation with increased levels of FSH and oestradiol and decreased levels of sex hormone binding globulin (SHBG) compared to a control group (82).

Many hypotheses have been put forward on the causes of precocious puberty in these girls but the underlying mechanisms remain unclear. Both central and peripheral mechanisms have been investigated with conflicting results. The first report on an association between CPP and international adoption, mentioned above, identified the extremely rapid catch-up growth as the most likely trigger for puberty (83).

In the following years, much credence was given to this hypothesis and improved socio-economic and nutritional conditions were thought to be the most likely underlying causes. This same hypothesis was put forward to explain the secular trend observed in the general population (25).

It was also speculated that increased levels of circulating insulin-like growth factor 1 (IGF-1) which have a facilitating effect on the gonads and downregulate neuropeptide Y expression following refeeding, could be key mechanisms implicated in the onset of precocious puberty, triggered by a rapid increase in height and adiposity following adoption (80). Studies *in vivo* have proved that NPY levels are implicated in triggering puberty (84).

In the following years, as knowledge advanced, increased levels of leptin and ghrelin, subsequent to an increased calorie intake, associated with changes in body composition and adipose tissue were advocated as important contributors (85, 86).

In 2012, Proos and Gustafsson reviewed the consequences of undernutrition and nutritional rehabilitation on the timing of pubertal onset and concluded that catch-up growth was very likely implicated in early pubertal development in cases of undernutrition in the prenatal and early postnatal period, whereas isolated late post-natal undernutrition alone did not seem to affect pubertal onset (87).

Prenatal and perinatal complications, such as low birth weight and intra-uterine growth retardation, are frequent in internationally adopted children (88), although data relative to pregnancy, and early life are often missing. Some variability depending on the country of origin should also be considered. Children from different developing countries present different relative risks of CPP, with a higher risk for adopted children from Africa and Latin America; no rise in the relative risk of CPP has been described instead for girls adopted from South Korea moving to Denmark and from China moving to North America (2, 10, 89). These differences may be explained by yet to be understood genetic factors and by environmental factors, e.g. different living conditions and dietary habits before adoption. Among the environmental factors, endocrine disrupting chemicals (EDCs) with estrogenic effects may play a role. In the last two decades, increasing attention has been given to the possible effects of exposure to EDCs during pre-natal and early post-natal life, and puberty (90). EDCs seem to be involved in changes in pubertal timing, although the complexity of exposure in real life makes it difficult to establish a direct causality (13, 14, 91–95). In a Belgian retrospective study on 145 patients, detectable concentrations of organochloride pesticides were found in foreign children, both adoptees and immigrants who were referred for CPP, whereas native Belgian children had undetectable serum levels (12). However, whereas it is easy to hypothesize an effect of these EDCs in the children who arrived in Belgium at a very early age, it is difficult to think of a significant effect on girls adopted at a later age who developed CPP within a few months of arrival.

Finally, emotional neglect and affective deprivation must be carefully considered when analysing plausible causes for CPP (31, 32, 96). Interestingly, an increased risk of CPP has been reported in adopted children but not in children migrating with their families from the same country of origin (2, 91–102). Genetic factors, intrauterine growth retardation, pre-adoption nutritional status, pre- and post-adoption growth patterns, as well as environmental exposures and psychological stress have been all put forward as possible triggers for precocious puberty in this population (100). Prior to adoption, significant stressors such as abuse and neglect that are known to affect brain structures are commonly found but may not always be communicated to the child’s doctor. Stressful nurturing conditions and insecure attachment to parents have also been commonly experienced by these children (26).

Frontline clinicians tend to focus on post-adoptive family experiences rather than on the adversities experienced prior to adoption, such as orphanage life, multiple foster care placements, and sexual abuse. However, several studies have investigated the connection between pre-adoption adversity and trauma and early onset puberty (101). A study by Noll et al. (102) found an association between childhood sexual abuse and early puberty. The research highlighted how survivors of sexual abuse may be at greater risk for psychosocial difficulties.

Interestingly, a high risk of CPP has been described for domestically adopted girls also. These observations refer to children being exposed to similar genetic and environmental factors, but with differences in living conditions before and after adoption that may have an effect. Therefore, emotional factors could be relevant for the early activation of the HPG axis and thus the onset of puberty, and possibly have a greater effect than other factors hypothesized so far.

## Adoption and exposure to trauma

The mechanisms that trigger precocious puberty in adopted children are still unknown (98). A high frequency of precocious puberty has been reported, as detailed above, in internationally adopted girls; this has led some authors to hypothesize a link with variables related to the country of origin and age of adoption (99). Genetic factors, intrauterine growth retardation, pre-adoption nutritional status, pre- and post-adoption growth patterns, as well as environmental exposures and psychological stress have been all put forward as possible triggers for precocious puberty in this population (100). Prior to adoption, significant stressors such as abuse and neglect that are known to affect brain structures are commonly found but may not always be communicated to the child’s doctor. Stressful nurturing conditions and insecure attachment to parents have also been commonly experienced by these children (26).

Frontline clinicians tend to focus on post-adoptive family experiences rather than on the adversities experienced prior to adoption, such as orphanage life, multiple foster care placements, and sexual abuse. However, several studies have investigated the connection between pre-adoption adversity and trauma and early onset puberty (101). A study by Noll et al. (102) found an association between childhood sexual abuse and early puberty. The research highlighted how survivors of sexual abuse may be at greater risk for psychosocial difficulties.

## Precocious puberty during the covid-19 pandemic: current data and hypotheses

In 2020, within a few months, the severe acute respiratory syndrome-Coronavirus-2 (SARS-CoV-2) virus responsible for coronavirus disease 2019 (COVID-19) caused a global pandemic, resulting in serious challenges for the health services of all countries (103). Most governments chose to introduce home quarantining (lockdowns) leading to sudden and radical changes in social interactions and in studying and working conditions (103). Many people’s diet and exercise patterns were disrupted and access to medical treatment was restricted (103, 104). Most children stopped going to school or doing leisure activities and attended online classes at home for several weeks. Many countries had more than one lockdown during which children stayed at home for long periods (103). Outdoor physical activity was prohibited or severely restricted (104, 105).

The repercussions of lockdowns on the physical and mental health of individuals appears to have been huge, and as yet has not been fully investigated (106, 107). In previous health crises such as SARS, Ebola and H1N1, the adverse effects observed in health workers, survivors and affected populations included depression, isolation, fear of being infected or infecting family members, post-traumatic stress, irritability, insomnia, anger and anxiety. Changes in nutritional habits and physical activity correlated to a marked deterioration in mental health (107).

Although children and adolescents infected by SARS-CoV-2 have a low risk of developing serious symptoms or critical illness, changes in lifestyle caused an increase in some endocrine diseases (108). For example, in Italy, outpatient treatment for suspected symptoms of CPP and EP increased dramatically in 2020 compared to the same time period in 2019 (105, 109) and the incidence of rapidly progressive precocious puberty (RPPP)/rapidly progressive early puberty (RPEP) also increased (109).

This phenomenon appears to be global (4, 110, 111) with cases of precocious puberty increasing dramatically in every country after lockdown restrictions were lifted (103, 105, 108, 109, 112–126). The first report of an increase in the number of outpatients with new-onset CPP from January to May 2020 was from the Meyer Children’s University Hospital in Florence, Italy, which reported a 2 fold increase compared with the same period in previous years. In addition, RPPP/RPEP were described more frequently than in previous years (109). In these patients, there were no significant differences regarding time between appearance of the breast bud and the diagnosis of CPP, with respect to previous years, but the age at presentation was lower with a more advanced Tanner stage at diagnosis, higher basal LH and E2 levels, higher peak LH after LHRH test and increased uterine length and ovarian volumes (109). Some of the girls showed a significantly accelerated progression rate of uterine length, and ovarian volumes. In both the CPP and CPP/RPPP groups, BMI increased significantly, and patients’ families reported an increased use of electronic devices (109). After a few months from this first report, the Bambino Gesù University Hospital of Rome (105) reported an increase of 108% in the number of consultations for suspected CPP with 215 diagnoses in 2020 compared to 87 in 2019 in females, whereas no difference was observed in male patients (105). This study did not find any differences in anthropometric parameters (105). Subsequently, other Italian groups (108, 118, 119, 122–124) reported similar results. Turriziani Colonna A et al. reported a high rate (48.9%) of RPPP in females with CPP during the COVID-19 outbreak, with an increased number of children at Tanner stages 3 and 4-5 at diagnosis (118). Interestingly, another Italian group described, in subjects with CPP, a later bedtime with higher rates of sleep disturbances, such as excessive somnolence, sleep breathing disorders, and sleep–wake transition disorders (119). The collaborative Italian study by Chioma et al. on a large study population of 490 children with CPP, transient thelarche, non-progressive PP, or early puberty, confirmed the higher number of CPP in females in 2020 compared to 2019 (p < 0.01), and whereas anthropometric and hormonal parameters were similar, a more prolonged use of electronic devices and a more sedentary lifestyle were reported (122). Interestingly, another Italian group did not find any differences in BMI SDS in females diagnosed with CPP in 2020 (median BMI SDS 0.11), who actually had a lower BMI compared to the girls who were diagnosed in 2019 (median BMI SDS 0.93) (108).

More recently an Italian group retrospectively evaluated clinical, biochemical and radiological data for 154 girls referred for disorders of precocious puberty, Early Puberty, isolated thelarche and isolated pubarche from January 2019 to April 2021 (123). The authors subdivided the observation periods into period 1 (before the lockdown: 1st January 2019 – 8th March 2020), and period 2 (the lockdown and the following months: 9th March 2020 – 30th April 2021). Period 2 was further subdivided into “restrictive lockdown period” (period 2.1: 9th March 2020 – 14th June 2020, in which schools were closed) and “less restrictive lockdown period” (period 2.2: 15th June 2020 – 30th April 2021) (123). Compared to period 1, the diagnoses of CPP increased significantly in period 2, without significant differences in auxological and hormonal data at diagnosis and rate of pubertal progression (123). The comparative analysis of sub-period 2.1 and 2.2 did not show any differences in auxological, laboratory and radiologic data, probably due to the reduced sample size (123). Interestingly, these authors showed that the percentage of girls who used personal computers and smartphones for more than 2 hours a day during lockdown was significantly higher in girls with CPP compared with the control group (123).

Finally, another Italian group, observing an increased proportion of consultations for suspected precocious puberty during the COVID-19 pandemic, showed a younger age at diagnosis and a lower bone age advancement than observed in pre-lockdown, suggesting a fast evolution of puberty (124).

Worldwide many research groups (103, 112–117, 120, 121, 125, 126) have reported similar results. For example, Acar et al. reported than double incidence of CPP in Turkish girls during the pandemic compared to the previous three-years; no significant increase in BMI SDS compared to the pre-pandemic period was reported (112), suggesting that factors other than increased BMI played a role in the development of CPP. Similar data have been reported in Korean (103), Turkish (115, 117), Indian (116), Chinese (113, 121), Spanish (114), Brazilian (120), Lebanese (125) and American girls (126) (**Table 1**).

**Table 1:** Studies evaluating the disorders of puberty development in females during and after COVID-19 pandemic.

Country	Age at diagnosis (years)	Pubertal disorders reported	Progression by B2	BMI SDS	Electronic devices use	Exercise time	Other findings (pandemic vs pre-pandemic)	Authors and references	
Italy	6.86 ± 0.61 (CPP)*(↓) 7.41 ± 0.61 (RPPP)	37 CPP 12 RPPP	↑	= (CPP) ↑ (RPPP)	↑	–	↑ basal and LH peak, ↑ estradiol, ↑ uterine length and ↑ ovary volume	Stagi S et al., 2020	(109)
Italy	7.33 ± 0.86	215°	–	=	–	–	No other data.	Verzani M et al., 2021	(105)
Italy	7.39 ± 0.84 (CPP) 7.19 ± 0.85 (NPPP) 7.03 ± 0.94 (TT) 8.64 ± 0.44 (EP)*	135 CPP 97 NPPP 64 TT 12 EP	–	= (CPP) = (NPPP) ↓ (TT) = (EP)	↑	↓	No differences in basal and LH peak, estradiol, uterine length and ovary volume	Chioma L et al., 2022	(122)
Turkey	7.80 ± 0.80	58 CPP	↑	=	–	–	23.4% showed menarche at diagnosis	Acar S et al., 2021	(112)
China	7.95 ± 0.77	191 CPP	–	=	↑	↓	No differences in basal and LH peak, estradiol. ↓ MKRN3, Ghrelin, GnRH, FSH	Chen Y et al., 2022	(121)
Brazil	7.70 ± 0.62	22 CPP	↑	=	–	–	↓ ovary volume	Oliveira Neto CP et al., 2022	(120)
Italy	7.59 ± 0.67	35 CPP	–	=	–	–	↑ basal LH and FSH, ↑ estradiol, ↑ sleep disorders	Umano GR et al., 2022	(119)
Italy	6.33 [1.16 - 7.10]	26 CPP	↑	= (CPP); ↑ at T1	–	–	BMI increased significantly at T1 with respect to T0	Turriziani Colonna A et al., 2022	(118)
India	8.20 ± 1.20	146 CPP	↑	=	–	–	No other data.	Mondkar SA et al., 2022	(116)
Turkey	7.70 ± 1.00*(↓)	145 CPP	–	=	↑	=	No differences in basal and LH peak, estradiol, uterine length and ovary volume	Yesiltepe Mutlu G et al., 2022	(115)
Turkey	7.50 ± 0.90 (CPP) 8.90 ± 1.10 (RPEP)	28 CPP 61 RPEP	↑	=^	–	–	No differences in basal and LH peak, estradiol, uterine length and ovary volume. ↑ early menarche cases.	Acinikli KY et al., 2022	(117)
Korea	–	–	–	–	–	–	Only epidemiological data. ↑ significative both male and female CPP	Choi KH et al., 2022	(103)
China	7.31 ± 1.00	58 CPP	–	↑	↑	↓	↓ vitamin D, ↓ serotonin, ↑ melatonin, ↑ leptin	Fu D et al., 2022	(113)
Italy	6.43 ± 1.50	17 CPP	=	–	↑	–	No differences in basal and LH peak, estradiol, uterine length and ovary volume.	Barberi C et al., 2022	(123)
Italy	8.20	45 CPP			–	–	Younger age at diagnosis; less bone age advancement at diagnosis	Goffredo M et al., 2023	(124)
Lebanon	7.27 ± 0.73	19 CPP	–	↑	–	–	Higher weight and more advanced bone age	Itali A et al., 2022	(125)
United States	7.6 ± 1.43	57 CPP	–	–	–	–	No differences in age at diagnosis, bone age, BMI SDS	Trujillo MV et al., 2022	(126)

F, females; CPP, Central Precocious Puberty; RPPP, Rapidly Progressive Precocious Puberty; NPPP, Non Progressive Precocious Puberty; TT, Transient Telarche; EP, Early Puberty; RPEP, Rapid Progressive Early Puberty. *Statistically significant respect to the prepandemic time; °referred for suspected precocious or early puberty; ^Increase of percentage of obese patients; ↑, increase/progression; ↓, reduction/decreas; =, no change.

An interesting paper by Fu et al. analysed the incidence and possible risk factors of female CPP during the COVID-19 Pandemic in China (113); 4281 girls were diagnosed with CPP between February and May 2020 (respectively 5.01 and 3.14 times more with respect to the same period in 2018 and 2019), and the authors concluded that the COVID-19 pandemic per se was a contributing factor for the general increase in the incidence of CPP (113). Interestingly, the authors reported a significant increase in CPP already in 2019 when the pandemic officially started in China and they highlighted that the BMI values of these girls were significantly higher than those in the control group. These data are reported also by the Lebanese group (125). Based on a questionnaire, the Chinese authors identified as high-risk factors for CPP the use of electronic devices for prolonged periods, less exercise time, higher BMI, vitamin D deficiency, frequent use of a night light, frequent use of adult cosmetics, consumption of fried food and processed meat, and exposure to second-hand smoke (113).

Sadly, there are scarce data on gender differences in the increase in cases of precocious puberty. However, males, unlike females, have an unclear onset of secondary sex characteristics, making it easy to miss signs of precocious puberty (127).

As previously stated, the timing of puberty is controlled by many environmental and nutritional triggers. It could be that infection with SARS-CoV-2 is capable of inducing puberty. The SARS-CoV-2 virus binds to the angiotensin-converting enzyme-2 receptor in the cranial nerves system especially around the olfactory bulb, where the concentration of GnRH neurons and GABAergic neurons is elevated (117) SARS-CoV-2 may also promote puberty onset by disrupting the blood-brain barrier or by direct interaction with neural pathways. NMDA receptors for example are stimulated by inflammatory cytokines and may be responsible for increased GnRH secretion (117, 128). However, many of the studies reported above did not involve patients who had been infected by SARS-CoV-2. Thus, it can be hypothesised that it was the chronic and prolonged stress related to living in during the pandemic that induced puberty in many of these patients. Stress may lead to the release of GnRH through certain neurotransmitters and neurons. Indeed, some data suggest that prolonged stress may accelerate puberty through NMDA, growth regulating factor 1 (GRF1), corticotropin releasing hormone (CRF), and γ-amino-butyric acid-A (GABA-A) receptors) in rats, and increased cortisol and catecholamines in mice (10, 129, 130) findings that are in part although not entirely in line with current knowledge in humans (77).

Furthermore, in some reports early puberty is associated with the use of methylphenidate that increases dopamine and norepinephrine, through transporter blockage, possibly triggering puberty, as the concentration in synaptic gaps increases (129). Previous studies have shown that CPP may be related to the environment (121), nutrition (131), and genetics (132).

Several studies have shown that CPP may be related to excess weight or obesity (111, 133, 134), possibly due to the influence of adipokines (particularly leptin and adiponectin) on the HPG axis (135, 136). High levels of leptin associated with an increase in BMI may stimulate the secretion of kisspeptin (137, 138). During the pandemic, long-term home quarantine, less time for outdoor exercise, and frequent fried food consumption caused rapid growth in children, often correlated with an increased BMI. However, few studies have reported an increase in BMI in the girls with CPP and RPPP compared to previous years (**Table 1**).

The use of electronic devices during the COVID-19 pandemic with reduced outdoor activity could also be implicated. Prolonged exposure to artificial light sources (blue light), including smartphones, tablets, and laptops, causes an inhibition of the secretion of melatonin (MT) (139–141); this hormone regulates the sleep-wake cycle and is inhibited by light (142–144). MT receptors are expressed in the hypothalamus, pituitary gland, and ovaries (145–147) and have a regulatory effect on the HPG axis, inhibiting the secretion of GnRH and thereby the initiation of puberty (148). Low melatonin levels affect the HPG axis, thereby accelerating the onset of puberty (149). In an observational study in schools in Cavriglia, Italy, the circulating levels of MT were 30% lower in children exposed daily to a television screen for one week compared to levels measured after a week of abstaining from TV (139). In another unpublished study conducted between October 2020 and March 2021, the salivary melatonin levels of 39 females diagnosed with CPP were significantly lower than those of the control group, and the effect was ascribed to the effects of light stimulation and electromagnetic fields (EMF) generated by electronic devices (150). Furthermore, serotonin, the precursor of MT, but not MT, was found to be significantly lower in girls with CPP compared with a control group in a Chinese study (113). These results may be related to a possible increase in the time spent on electronic devices. The same authors (113) investigated the possible effect of other factors, such as the exposure to exogenous oestrogen and reported a higher incidence of CPP in rural areas than in urban areas of China (113). According to a Korean study (103, 151), the rate of overweight and obese children who presented with precocious puberty in 2020 was significantly higher than in 2019, even if more prevalent in males leading the authors to speculate that obesity, fast food consumption, and the consumption of growth-related health functional foods could be factors accelerating the onset of puberty (152). Endocrine-disrupting chemicals may be another factor. Since social distancing and hygiene precautions led to a rapid increase in the use of disposable items such as plastic, vinyl and CPP (153). These chemicals are known to be factors facilitating puberty.

Finally, stressful life events may play an important role in determining menarcheal age (154, 155). The SARS-CoV-2 pandemic may have acted as a major stressor especially among children and adolescents (156, 157). Lockdowns, school closures and the need for social distancing were certainly stressful for children and led to less exercise, less healthy eating and longer periods of time at home in front of screens (158). In addition, the anxiety and financial concerns of parents, along with fears of becoming ill and higher exposure to family violence during the lockdown may have caused stress in children (159–161).

## COVID-19 as an environmental stressful factor

Lockdowns associated with the Covid-19 pandemic may have enhanced the impact of factors that interfere with the timing and rhythms of puberty. As mentioned above the incidence of precocious puberty, precocious menarche and accelerated puberty noticeably increased during the lockdown period (105, 109, 112, 122). In general, a higher incidence of depression, anxiety and stress was also reported (162, 163). The authors hypothesize that higher stress levels and changes in behaviour such as an increased use of electronic devices may have contributed to the rise in the incidence of CPP (109, 122). Certainly, children and adolescents experienced a stressful period during a very sensitive and vulnerable period of their lives. However, these aspects have not been well defined and documented in the Literature. Furthermore, in countries, such as Italy, disruption to daily life continued well after the initial lockdown.

## The psychological consequences of precocious puberty

Puberty is a crucial and sensitive phase of human development that leads to sexual maturity. The complex processes of biological transformations related to physical and sexual maturation triggers a series of physical and biological changes that throughout adolescence affect psychological and social development. The effects of physical changes on the adolescent body are evident and exceptional, and affect social behaviour (164), perception of the self and how an individual is viewed and treated by their peers and adults.

In addition to genetic and hormonal factors, the timing and speed of physical maturation are strongly influenced by factors related to the context in which development takes place. Such factors include the specific geographical setting, socio-economic status, the ethnic group to which a person belongs, and also the person’s physical health (165).

During this sensitive developmental phase, the onset of a disease or a significant physical change represents additional stress which may have a significant impact on many areas of an adolescent’s life (166). An individual’s ability to adjust to the changes brought about by puberty can be compromised if pubertal changes are not in accordance with maturational norms at a given age within their peer group. Adolescents who experience earlier pubertal maturation are at a heightened risk for psychopathology in adolescence (167).

For successful adaptation, it is important that the adolescent has sufficient personal and family resources to help him or her navigate the transitional period to adulthood.

The psychosocial consequences of early puberty have a strong correlation with the environment. Environments that are stressful, for example because of conflictual relationships with peers and family or because of socio-economic problems such as delinquency and scarce educational opportunities, increase the risk of mental health problems (168).

Adolescents with CPP have a greater need of parental support during pubertal transition to guide and prepare them to cope with the changes and different experiences associated with these transformations. Although early maturation appears to be associated with a greater emotional distance between adolescents and their parents (46), parent-adolescent secure attachment appears to be a protective factor that plays a crucial role in preventing distress and maladjustment. In addition, perceived parental attachment moderates the relationship between early maturation and behaviour in adolescents (169). An adolescent who perceives parents as a source of psychological security, is able to share and communicate better his/her difficulties, and to seek advice from parents. Thus, if adolescents with precocious pubertal development do not perceive their parents as available and responsive, they are more likely to exhibit problematic behaviour. It has also been shown that early experience influences age at reproduction more in females than males (5).

## Lessons from the COVID-19 pandemic, change of paradigm and conclusions

It is reasonable to hypothesise that in adopted and “covid-19 pandemic” children with precocious puberty a common trigger is present. Until recently, it was thought that the cause of CPP in adopted children was mainly improved nutrition leading to a rapid weight and height catch-up growth, coupled with improved psychological wellbeing following adoption. During the pandemic several factors came into play that may have contributed to inducing or accelerating pubertal development such as increased weight, reduced opportunity for exercise, increased use of technology, and changes in diet and sleeping habits.

It is our belief, however, that the main driver behind the increased incidence of CPP during the lockdown period was the environment of “fear and stress” in which many children lived at this time. Normal social contact was impossible (even when children returned to school, masks and other PPE were often required) and many of the adults in daily contact with children suffered from anxiety about becoming ill, money and other problems. Furthermore, problems within families might have exacerbated due to the close contact of family members.

Stress related to a new environment may also be a factor in the high incidence of CPP in adopted children. Like children in lockdown, children adopted after infancy have recent memories of their previous lives. Despite their new and loving parents, adopted children may recall more than currently understood; learning to trust a new family and adapt to life in a country with different nutritional habits, culture, and lifestyle is likely to be stressful. This phenomenon requires further attention and understanding and has not been thoroughly studied. In fact, specific references are missing from the Literature.

While the Literature suggests that early life psychosocial stress correlates with early sexual maturation there are some caveats. It is important to highlight that these data are from WEIRD (Western, Educated, Industrialized, Rich and Democratic) populations. Data from traditional societies call into question the strong link between paternal investment and child wellbeing: in fact, it is very unlikely that the so called nuclear family is representative of the kind of family most often present throughout human evolutionary history (170). This does not mean that paternal or maternal attachment are not important factors in reducing a child’s mortality and morbidity risks, rather, it underscores the extraordinary complexity of the social environment that has characterized childhood throughout human evolution and that is still represented cross-culturally, with mothers, fathers, grand-parents and access to supportive kin’s networks influencing the extent to which children are exposed to threat and deprivation and how the stress system has evolved in adaptive ways (171). In fact, from an evolutionary neuro-biological point of view, variations in the timing of sexual maturation within a species are part of a strategy that depends on internal and external environmental conditions (5). We hypothesize that in a society where there are good living conditions, a general threat to life, such as that presented by the SARS-CoV-2 pandemic, may trigger early pubertal maturation to favour early reproduction.

Finally, we would recommend families, in order to reduce the risk of precocious puberty, to maintain an appropriate lifestyle including healthy eating and sufficient movement, to reduce as much as possible the time spent in front of electronic devices for their children, to be cautious bringing worries and money problems to their children, to be careful and respectful of any fears, to leave time to adopted children to adapt to new families and lives, to interact with teachers and the school environment if any concern arises.

## Author contributions

MS and SS conceptualized, wrote paragraphs, revised and obtained approval from all authors. DP gave substantial contribution to the writing of the manuscript and draw **Figure 1**. RR, MP, TD, CL, VF, and DR performed the research of the Literature, and drafted sections of the manuscript, revised and approved its contents. All authors contributed to the article and approved the submitted version.
